# Multidisciplinary Treatment for Colorectal Peritoneal Metastases: Review of the Literature

**DOI:** 10.1155/2016/1516259

**Published:** 2016-12-26

**Authors:** Shaobo Mo, Guoxiang Cai

**Affiliations:** ^1^Department of Colorectal Surgery, Fudan University Shanghai Cancer Center, Shanghai 200032, China; ^2^Department of Oncology, Shanghai Medical College, Fudan University, Shanghai 200032, China

## Abstract

Peritoneum is one of the common sites of metastasis in advanced stage colorectal cancer patients. Colorectal cancer patients with peritoneal metastases (PM) are traditionally believed to have poor prognosis, which indicates it is of no value to adopt surgical treatment. With the advancement of surgical techniques, hyperthermic intraperitoneal chemotherapy (HIPEC), and multidisciplinary treatment in recent years, the cognition and treatment strategies of colorectal peritoneal metastases (CPM) have changed dramatically. In terms of prognosis, CPM under the palliative systemic treatment shows an inferior outcome compared with nonperitoneal metastasis. Nevertheless, some CPM patients amenable to the complete peritoneal cytoreductive surgery (CRS) combined with HIPEC may achieve long-term survival. The prognostic factors of CPM comprise peritoneal carcinomatosis index (PCI), completeness of cytoreduction score (CC score), the presence of extraperitoneal metastasis (liver, etc.), Peritoneal Surface Disease Severity Score (PSDSS), Japanese peritoneal staging, and so forth. Taken together, literature data suggest that a multimodality approach combining complete peritoneal CRS plus HIPEC, systemic chemotherapy, and targeted therapy may be the best treatment option for PM from colorectal cancer.

## 1. Introduction

Peritoneal spread is common in advanced stage colorectal cancer patients and was reported in the past to be associated with a poor prognosis [1–3]. Approximately 5.0% of colorectal cancer patients present synchronous peritoneal metastases (PM) and about 19.0% may manifest metachronous disease [4, 5]. PM is often accompanied by distant metastasis of other organs, such as the liver and lungs. It has been reported that 88% of colorectal peritoneal metastases (CPM) had other concomitant distant metastases [6]. PM is traditionally perceived as the advanced manifestation of colorectal cancer, with a median survival of 5–7 months, which hardly has the healing possibility and the value of surgical treatment [1, 7, 8].

With the purpose of seeking more effective treatments for CPM, myriads of approaches have been undertaken over the past decades. Recently, with the accumulation of massive researches on CPM, there is a better understanding of the prognosis of CPM and prognostic influence factors [9]. Moreover, the attitude towards the therapeutic strategies for CPM has changed tremendously with the update of the concept of multidisciplinary treatment and advances in surgical techniques and hyperthermic intraperitoneal chemotherapy (HIPEC) [10, 11]. Therefore, the therapeutic efficacy of PM has been greatly improved, achieving better long-term survival in patients with PM from colorectal carcinoma [12].

In this review we commit to exploring the recent advances in multidisciplinary treatment for CPM.

## 2. Risks for Development of CPM

Defining risk factors for the development of CPM is needed to better select patients at high risk for developing PM, which consequently might benefit from intensified adjuvant treatment regimens.

In the population-based study by Segelman et al. [3], independent predictors for developing metachronous CPM were colonic cancer, in particular right-sided, stages T3-T4 tumor, lymph node statuses N1-N2, fewer than 12 lymph nodes harvested, emergency procedures, and nonradical resection of the primary tumor (R1 or R2). From a prospectively expanded single-institutional database with 2406 consecutive patients with colorectal cancer (CRC), clinical and histological data were analyzed for independent risk factors, the results of which demonstrated that age <62 years, N2-status, T4-status, and location of the primary in the left colon or appendix were independent risk factors for the development of metachronous CPM [13].

The results of study by Lemmens et al. [2] showed that the risk of synchronous CPM was increased in case of advanced T stage, advanced N stage, poor differentiation grade, younger age, mucinous adenocarcinoma, and right-sided localization of primary tumor. In the study by Jayne and coworkers [1], 53% of the patients with synchronous PM had a rectal cancer and as for the remaining patients, the primary tumor was located in the left, transverse, and right colon in 25%, 7%, and 16%, respectively. According to the study by Sadahiro et al. [14], the primary tumor was most often located in the right colon (*p* = 0.02), with significantly more lymph node metastases in patients with synchronous PM (*p* < 0.001), while the primary tumor was mostly located in the left colon (34%) in the study of Mulsow et al. [15].

## 3. The Prognosis and Prognostic Factors of CPM

Recent clinical studies show that, receiving palliative systemic treatment alone, patients with CPM have poorer prognosis than those diagnosed with nonperitoneal metastasis. However, the therapeutic approach combining cytoreductive surgery with HIPEC and systemic treatment can achieve long-term survival in appropriate patients with PM from colorectal origin. Furthermore, peritoneal carcinomatosis index (PCI), completeness of cytoreduction score (CC score), liver metastasis, and other related factors are the prognostic influence factors of CPM patients.

### 3.1. The Prognosis of CPM

A pooled analysis of North Central Cancer Treatment Group Phase III Trials N9741 and N9841 enrolled 2,095 patients with metastatic colorectal cancer (mCRC) and 364 patients with CPM included, receiving palliative chemotherapy (5-fluorouracil, oxaliplatin, or irinotecan). The results of the research showed that patients with CPM shared a significantly shorter median overall survival (OS) (12.7 versus 17.6 months, *p* < 0.001) and progression-free survival (PFS) (5.8 versus 7.2 months, *p* = 0.001) compared with nonperitoneal mCRC patients [6]. Similar conclusion can also be drawn in the CAIRO study (previously untreated mCRC patients were treated with chemotherapy) and the CAIRO2 study (previously untreated mCRC patients were treated with chemotherapy and targeted therapy). Klaver et al. [16] reported that no matter how chemotherapy is used alone or combined with targeted therapy, the median OS in patients undergoing CPM were markedly lower than that in patients with mCRC without peritoneal involvement.

CPM patients under the palliative systemic treatment were reported to be connected with poor outcomes, but the prognosis of CPM patients will change noticeably with effective surgical therapy combined with HIPEC. Elias et al. [17] reported the results of their research conducted in French Gustave Roussy Hospital. This research recruited 287 colorectal cancer patients with liver metastases on whom a complete (R0) hepatic lesions resection was performed and 119 cases with PM undergoing peritoneal cytoreductive surgery (CRS) plus HIPEC between 1993 and 2009, excluding patients presenting both liver metastases and PM. The results showed that there were no statistical differences between the liver metastases group and the PM group in 5-year OS rates (38.5% and 36.5%, resp.; *p* > 0.05). A study from Australia has also come to the similar conclusion, whose results revealed that the 5-year OS rates of colorectal cancer patients with liver metastases or PM treated with R0 resection of the liver disease or CRS plus HIPEC, respectively, were 33.3% and 32.1% (*p* > 0.05) [18].

### 3.2. The Prognostic Factors of CPM

#### 3.2.1. Peritoneal Carcinomatosis Index

Peritoneal carcinomatosis index (PCI), a scoring system that quantifies the extent of carcinomatosis, has been recognized as one of the most important prognostic indicators for the long-term outcomes of CPM patients [19–22]. The size and distribution of peritoneal deposits were recorded using the PCI system as described by Glehen et al. [12]. The abdomen is divided into thirteen regions: central region (0), right upper region (1), epigastrium region (2), left upper region (3), left blank region (4), left lower region (5), pelvis region (6), right lower region (7), and right blank region (8), and the small bowel is divided into four: upper jejunum region (9), lower jejunum region (10), upper ileum region (11), and lower ileum region (12) [12, 23]. Each one is assigned a lesion-size (LS) score of 0 to 3, which would be representative of the largest implant lesion visualized. LS-0 stands for no tumor seen, LS-1 indicates implants <0.25 cm, LS-2 indicates implants between 0.25 and 5 cm, and LS-3 indicates implants >5 cm or a confluence of disease [12, 23]. PCI score is a final numerical score of 0–39 [12, 24]. One recent study conducted by Faron et al. (2016) demonstrated a perfect linear relationship between the PCI and OS [25]. A survival analysis according to the PCI indicated that the 5-year OS rate was prominently higher in CPM patients with a low PCI (<10) than in patients with a PCI ranging from 10 to 20 or a PCI of more than 20 (53%, 23%, and 12%, resp.; *p* < 0.001) [26]. Besides, Elias et al. [10] reported a 44% of 5-year OS rate with a PCI score of 6 or less and 7% with a score greater than 19. PCI also influences the likelihood of complete cytoreduction. Most scholars assert that CPM patients with PCI >20 have poor oncological outcomes, thus unsuitable for the extensive peritoneal CRS.

#### 3.2.2. Completeness of Cytoreduction Score

Completeness of cytoreduction score (CC score) reflects the thoroughness degree of peritoneal CRS. CC score is calculated according to the maximum diameter of residual tumor after surgery: CC-0 indicates no residual tumor, CC-1 stands for the maximum diameter of residual tumor <2.5 mm, CC-2 stands for the maximum diameter of residual tumor between 2.5 mm and 2.5 cm, and CC-3 stands for the maximum diameter of residual tumor > 2.5 cm [27]. For colorectal cancer, a complete cytoreduction score includes both CC-0 and CC-1: CC-0 indicates that all visible tumors are removed completely, and CC-1 presents that only a very small amount of residual tumors that are expected to be eradicated by the HIPEC remain not resected [28]. Complete CRS is not only an important prognostic influence factor but also a necessary requirement for long-term benefit in the management of PM. A French multicentre trial included 523 patients who had undergone operation from 23 centers in four French-speaking countries between 1990 and 2007, the results of which showed remarkably better survival in patients with complete CRS than in those with incomplete CRS [10]. Huang et al. [29] found that CRS plus perioperative intraperitoneal chemotherapy can be safely performed to provide encouraging survival benefits for patients with CPM. Hence, CC-0 and CC-1 have been recommended to be the standard for CRS by most researches. Conversely, CC-2 and CC-3 surgery should not be recommended presently.

#### 3.2.3. Liver Metastasis

It is still controversial when it comes to the prognosis of CPM patients with liver metastasis. On the one hand, the concomitant liver metastasis is a poor prognostic factor. Related researches showed that the prognosis of CPM patients with liver metastasis was conspicuously worse than that of patients with CPM [30]. Elias et al. [10] reported in a multivariate analysis that even if a complete CRS could be obtained with effort, a resectable liver metastasis during the CRS was still a negative prognostic indicator for CPM patients. Glehen et al. [12] confirmed that CPM patients with simultaneous liver metastasis were linked with worse outcomes. On the other hand, a systematic review analyzed the prognosis of colorectal liver metastasis patients with extrahepatic metastasis undergoing surgical resection, the result of which showed that, with the liver resection plus peritoneal CRS for the CPM patients, the 5-year OS rate of 25% can be materialized [31]. Kianmanesh et al. [32] reported that there were no distinct discrepancies as for the survival rate between patients who underwent CRS plus HIPEC for PM alone (including the primary resection) and those who had liver metastasis resection (median survival, 35.3 versus 36.0 months, *p* = 0.73). Some studies have shown that CPM patients with localized PM (PCI < 12) and limited liver metastases (less than 3 metastatic lesions) may benefit from liver metastasis resection and peritoneal CRS [30]. Thus, some authors propose to consider intensive therapy only in patients with PCI <12 and <3 liver metastases [30, 33, 34].

#### 3.2.4. Other Related Factors

The Peritoneal Surface Disease Severity Score (PSDSS) was put forward by The American Society of Peritoneal Surface Malignancies [35]. PSDSS consists of 3 prognostic categories: (1) clinical symptoms, (2) extent of peritoneal dissemination (PCI determined on a computed tomographic scan), and (3) primary tumor histology, each of which is subcategorized according to severity [36, 37]. PSDSS can be performed when patients are diagnosed with CPM without the need for intraoperative staging [35]. Clinical analyses testified that PSDSS was an independent prognostic indicator concerning survival not only for patients who underwent a complete CRS and HIPEC, but also for patients treated with systemic chemotherapy without CRS [36, 38–40]. However, although PSDSS is a very interesting system as a recent proposition, it is not used in a common practice and not a rule to be as equivalent as PCI.

The Japanese Society for Cancer of the Colon and Rectum (JSCCR) classifies PM into three subgroups: P1 indicates metastases only to adjacent peritoneum, P2 stands for a few metastases to distant peritoneum, and P3 represents numerous metastases to distant peritoneum. Relevant researches certified that different P stage was significantly associated with different prognosis [5, 41].

## 4. Treatment for CPM

### 4.1. Peritoneal CRS

Colorectal peritoneal metastatic lesions and organs involved should be surgically removed during CRS. Apart from peritoneum, greater omentum, lesser omentum, gallbladder, appendix, and ovaries may often be resected, sometimes part of small intestines, rectum and sigmoid colon, uterus, spleen and distal stomach, and so forth as well. Surgeons should attempt to remove all the visible tumors to obtain a complete cytoreduction during peritoneal CRS [34]. Based on current literature, the principle of the CPM resection should meet both the criteria of oncology and the criteria of organ function. The criteria of oncology consist of 3 aspects: (1) PCI < 20, (2) surgical resection of all the peritoneal metastases or the maximum diameter of residual tumor <2.5 mm (CC-0 and CC-1), and (3) R0 resection of the primary tumor and nonperitoneal metastases (liver, lung, or ovary). The criteria of organ function encompass 2 facets: (1) age < 75 and KPS > 70 and (2) small intestinal mesentery without severe contracture and no severe small intestinal obstruction. The preoperative assessment of the CPM surgery should include the oncology and the functional evaluation. The oncology evaluations are as follows: (1) tumor antigen (CEA, CA19-9, and CA125), (2) imaging evaluation of peritoneal metastases (enhanced multislice computed tomography (CT) scans plus multiplanar reconstruction, enhanced magnetic resonance imaging (MRI), or positron emission tomography-computed tomography (PET-CT) of abdomen and pelvis), and (3) imaging evaluation of primary tumor and extraperitoneal metastasis. The functional evaluations include gastrointestinal dynamic contrast examination or enhanced CT of the abdomen and pelvis performed to figure out whether there are multisegmental small intestinal obstructions due to small intestinal mesentery contracture.

### 4.2. HIPEC

HIPEC refers to intraoperative delivery of chemotherapic agents in a recirculating perfusion of the abdominal cavity under hyperthermic conditions [42]. In addition to the traditional superiority of intraperitoneal chemotherapy (abdominal local high concentration, low concentration of peripheral blood, and mild systemic toxicity), HIPEC also has the following advantages: (1) the direct killing effect of hyperthermia, (2) the chemotherapy enhancement effect of hyperthermia, and (3) mechanical flushing effect of irrigation. It can be performed in a closed or open method, before or after surgery. The open method makes it more uniform in distribution of heat and chemotherapy drugs, but the operation is relatively inconvenient and heat emission exists. For the closed method, it can reduce the exposure of the medical personnel to chemotherapy; its performance is relatively simple; and the increased abdominal pressure may contribute to the peritoneal permeation of the chemotherapy drugs, whereas the problem is that heat and drugs are not uniformly allocated. At present, there is no gold standard for the chemotherapy drugs used in HIPEC. The American Society of Peritoneal Surface Malignancies (ASPSM) recommends patients to undergo 40 mg mitomycin intraperitoneal perfusion during 90 min at 42°C [43]. Elias et al. [44] from French Gustave Roussy Cancer Campus also reported a series of patients treated with oxaliplatin in the perfusate at 460 mg/m^2^ in 2 L/m^2^ during 30 minutes at 43°C and later reported another protocol with oxaliplatin 360 mg/m^2^ plus irinotecan 360 mg/m^2^ in 2 L/m^2^ of dextrose 5%. During the same time, patients received an intravenous administration of 5-fluorouracil (400 mg/m^2^) and leucovorin (20 mg/m^2^).

### 4.3. Systemic Chemotherapy

CPM patients have a poor prognosis, and the median survival without chemotherapy is about 6 months [1, 7]. In terms of systemic chemotherapy, there is no difference between CRPC patients and other nonperitoneal metastasis patients, but the effect of systemic chemotherapy is worse in the former compared with that in the latter. In the past few years, new drugs have been introduced into systemic chemotherapy. The application of targeted therapy drugs such as bevacizumab and cetuximab further improves the efficacy of peritoneal metastatic carcinoma. In the retrospective study, receiving chemotherapy in combination with targeted therapy could significantly prolong the OS of CPM patients to 18.2 months, the effect of which is still worse than that of the patients with liver or lung metastases yet [45].

## 5. Multidisciplinary Treatment Strategy for CPM

Complete peritoneal CRS plus HIPEC and systemic treatment (including chemotherapy and targeted therapy) are currently the best modality of multidisciplinary treatment for CPM patients. During CRS, all the visible tumors should be removed to obtain a complete peritoneal CRS. In addition to the cytoreductive surgery, HIPEC is administered to eradicate the tiny and microscopic peritoneal diseases. Systemic chemotherapy is always significant since PM is often part of systemic metastasis. The combined application of these three aspects will probably improve the prognosis of colorectal peritoneal metastatic carcinoma.

According to the National Comprehensive Cancer Network (NCCN) guidelines, combined peritoneal CRS and HIPEC have not been routinely recommended for CPM patients. Nonetheless, the NCCN guidelines also recommend to carry out more well-designed clinical trials. In contrast, the European Society for Medical Oncology (ESMO) guidelines hold more positive attitude towards it. The ESMO guidelines recommend that CPM patients with low PCI should be treated with surgery plus HIPEC when a complete CRS can be obtained. In Japan, a complete CRS has been recommended for patients with P1 phase CPM as well as P2 phase CPM if technically possible [5]. In China, combined peritoneal CRS and HIPEC have also been recommended by experts for CPM patients with PCI <20.

Randomized clinical trials have confirmed that combined peritoneal CRS and HIPEC plus systemic chemotherapy is superior to palliative systemic therapy for CPM patients (the median survival, 22.3 and 12.6 months, resp., *p* = 0.032) [19]. The 5-year survival was 45% for those patients in whom a complete CRS was achieved [46]. The superiority of combined peritoneal CRS and HIPEC plus systemic chemotherapy can also be clarified in a retrospective study which compared patients undergoing CRS plus HIPEC with comparable controls treated with palliative chemotherapy during the same period but who did not benefit from CRS plus HIPEC because the technique was unavailable in the center at that time. The 5-year OS rates of patients undergoing combined peritoneal CRS and HIPEC plus systemic chemotherapy were significantly higher than that of the patients with palliative systemic therapy (51% versus 13%, resp.) [47].

For CPM patients with PCI >20, peritoneal CRS is not suitable for them currently. However, with the appearance of more effective chemotherapeutic and targeted drugs, it is possible to achieve the reduction of PCI by effective chemotherapy, thereby creating the possibility for further surgery. Because of the high risk of postoperative recurrence of peritoneal metastasis, it is held that CPM patients with PCI <20 may benefit from the modality of perioperative chemotherapy.

## 6. Controversial Issues and Latest Progress

### 6.1. Diffusion-Weighted MRI

Preoperative MRI and CT of the abdomen and pelvis play a critical role in the assessment of the extent of peritoneal and visceral disease in patients being considered for CRS and HIPEC for CPM [48]. In comparison with CT, MRI uses different types of image contrast to create images that are more sensitive for detection of peritoneal lesions [49–51]. With major refinements in hardware and software, functional MRI such as diffusion-weighted MRI (DWI) has become technically possible. Various tumors actuate restricted diffusion of water protons which can be evaluated by DWI, resulting in an area of altered signal on DWI [48]. The sensitivity of DWI for depicting peritoneal tumor has opened the gate to potential advancements in detecting disseminated peritoneal metastatic disease. Recently, there have been several very interesting researches regarding DWI [52]. In 2012, Low and Barone [53] calculated the PCI based on DWI before surgery in 35 patients with peritoneal metastatic disease. Compared to surgical-site findings, the overall sensitivity and specificity were 88% and 74%. Besides, Espada et al. [54] developed a scoring system with a diagnostic accuracy of 91% by evaluating DWI for detection of PM in 34 patients. In a more recent report [48], preoperative DWI and CT scanning to determine the PCI were performed in 22 patients with PM undergoing CRS, where DWI demonstrated predictive accuracy of 91%, 83%, and 94% in overall category, low to moderate tumor burden (PCI ≤ 20), and large tumor burden (PCI > 20), compared with CT with a corresponding predictive accuracy of 50%, 50%, and 44%. Of note, it has been reported in 2012 that DWI and 18 fluoro-deoxy-glucose positron emission tomography-computed tomography (18FDG-PET/CT) showed similar high accuracy in diagnosing peritoneal metastatic carcinoma [55]. Furthermore, DWI appears more sensitive than PET/CT in the supramesocolic area; nevertheless, the limitation of lower sensitivity in the detection of small implants still exists in DWI [55]. Interestingly, a prospective comparison of CT, PET/CT, and whole body DWI in 32 patients conducted by Michielsen et al. [56] in 2014 noted an accuracy for detection of peritoneal disease of 91% on DWI compared with 75% on CT and 71% on FDG-PET/CT, with surgical reference standard in all cases. It is desirable to improve the detection and characterization of peritoneal implants by the simultaneous use of 18FDG-PET and DWI, which remains to be confirmed by future studies [57, 58].

### 6.2. Fluorescence Imaging

Intraoperative fluorescence imaging- (FI-) guided surgery is an emerging technology in the war against cancer that has been proved to improve tumor detection in different tumor types [59–61]. Among the different probes used in identifying neoplasm, indocyanine green (ICG) has been introduced as a safe and useful indicator [61]. Until now, only a few clinical studies have analyzed the potential role of FI taking in PM [62]. Liberale et al. [62] demonstrated in a pilot study that sensitivity and specificity of ICG-FI in patients with nonmucinous CPM were 87.5% and 100%; contrarily, among the mucinous tumors, the sensitivity of ICG-FI was 0%. Unexpectedly, in 4 out of 14 patients (29%), additional PM that were not found using visualization and palpation were detected in the surgery modified by intraoperative ICG-FI [62].

### 6.3. Catumaxomab

Catumaxomab is a trifunctional monoclonal antibody with a mouse-derived anti-EpCAM Fab (fragment antigen-binding) region and a rat anti-CD3 Fab [63]. Catumaxomab antineoplastic activity has been confirmed in vitro, notably in malignant ascites, resulting in a decreased rate of EpCAM plus cells and the release of proinflammatory cytokines (interferon-*γ*, tumor necrosis factor-*α*, interleukin- (IL-) 2, and IL-6), which enables tumor cells of PM to be specifically identified by catumaxomab via the anti-EpCAM-binding site [64, 65]. Therefore, intraperitoneal catumaxomab therapy represents a targeted immunotherapy against PM [66]. A randomized phase II/III trial was carried out in 258 patients with symptomatic malignant ascites secondary to EpCAM + carcinomas, to assess the efficacy and safety of intraperitoneal catumaxomab treatment [67]. In this study, patients were randomly assigned to paracentesis plus intraperitoneal catumaxomab or paracentesis alone. The primary endpoint was puncture-free survival used to evaluate the efficacy of intraperitoneal administration of catumaxomab. Puncture-free survival was significantly longer in the group treated with catumaxomab than the control group (46 versus 11 days, *p* < 0.0001). Median overall survival was similar between the two groups (72 versus 68 days, *p* = 0.0846).

### 6.4. Systematic Second-Look Surgery and HIPEC

Systematic second-look surgery and HIPEC for CRC patients considered to be at high risk of developing PM have aroused a growing interest among the scientific community. CPM is generally diagnosed in its advanced phase because of the late symptoms onset, low sensitive imaging techniques, and tumor markers. Therefore, the concept of early intervention has emerged. Second-look surgery and HIPEC in patient with a high risk of developing PM treated with curative surgery for CRC have been proposed, but again this would be limited to a small group of patients [68]. In the prospective study, Elias and colleagues reported 29 selected patients at high risk of developing PM without any sign of recurrence on imaging studies who underwent second-look surgery 13 months after resection of the primary tumor. In 55% of patients, persistent adenocarcinoma was documented. In patients with documented disease at second look 9 of 16 patients were disease-free at 27 months of median follow-up [69]. Besides, patient with or without macroscopic PM at second-look surgery treated with CRS and HIPEC developed a low rate of peritoneal recurrences (17% at a median follow-up of 30 months) [70]. One ongoing prospective randomized clinical trial is designed in France. All patients at risk will receive the gold standard adjuvant systemic chemotherapy with FOLFOX 6 over 6 months. Patients with a negative follow-up will be randomly assigned to surveillance or second-look laparotomy and HIPEC. The object of the clinical trial is to evaluate the peritoneal recurrence rate for 3 years.

### 6.5. The Necessity of HIPEC

CRS plus HIPEC is increasingly being used for the treatment of PM of colorectal origin. However, it is still controversial whether HIPEC is the indispensable cornerstone of the best modality of multidisciplinary treatment for CPM [71]. It is not yet possible to evaluate the morbidity and mortality rates related to HIPEC alone independently of CRS, as both procedures are performed jointly during the same surgery. Only those patients who undergo CRS can receive HIPEC. The published mortality rates in CRS + HIPEC range from 0 to 11% (mean 4–6%) [71]. Survival rates are reported better in patients who undergo CRS + HIPEC versus those who receive systemic chemotherapy [72]. Seeking to elucidate the true role of HIPEC in the context of radical combined therapy, a multicentre, randomized, open-label study currently in phase III (PRODIGE 7) was designed with parallel groups and two treatment arms comparing CRS + HIPEC + systemic chemotherapy before or after surgery versus CRS + systemic chemotherapy before or after surgery. The final collection date for primary outcome measure was completed in December 2015. Once the results of this study are achieved, we should have considerably more evidence regarding the efficacy of this treatment modality [71].

## 7. Conclusion

Complete peritoneal CRS plus HIPEC and systemic treatment (including chemotherapy and targeted therapy) might be the best modality of multidisciplinary treatment for CPM. Patients appropriate for aggressive therapy undergoing complete peritoneal CRS plus HIPEC and systemic treatment can get better long-term outcome. PCI and CC score are important prognostic indicators of CPM patients. More well-designed clinical trials are in terrible need to figure out the best multidisciplinary treatment modality for CPM.
